# Master protocols in vocal biomarker development to reduce variability and advance clinical precision: a narrative review

**DOI:** 10.3389/fdgth.2025.1619183

**Published:** 2025-06-27

**Authors:** Ayush Kalia, Micah Boyer, Guy Fagherazzi, Jean-Christophe Bélisle-Pipon, Yael Bensoussan

**Affiliations:** ^1^ USF Health Voice Center, Department of Otolaryngology-Head & Neck Surgery, University of South Florida, Tampa, FL, United States; ^2^ Deep Digital Phenotyping Research Unit, Department of Precision Health, Luxembourg Institute of Health, Strassen, Luxembourg; ^3^ Department of Bio-ethics, Faculty of Health Sciences, Simon Fraser University, Burnaby, British Columbia, Canada

**Keywords:** voice, biomarkers, master protocol, vocal biomarkers, digital biomarkers, voice AI, speech biomarkers

## Abstract

**Introduction:**

Vocal biomarkers, defined as acoustic or linguistic features extracted from voice samples, are an emerging innovation in medical diagnostics. Utilizing artificial intelligence, machine learning, or traditional acoustic analysis, vocal biomarkers have shown promise in detecting and monitoring conditions such as respiratory disorders and cognitive impairments. Despite their potential, the lack of standardized protocols for data collection and analysis has limited their clinical applicability.

**Objectives:**

This review assesses the current state of research on developing a master protocol for vocal biomarkers, identifying key aspects essential for reducing variability across studies. It also explores insights from digital biomarker research to inform the creation of a standardized framework for vocal biomarker development.

**Methods:**

A narrative review was conducted by searching PubMed for literature on vocal and digital biomarker development. Articles were evaluated based on their proposed frameworks and recommendations for addressing methodological inconsistencies.

**Results:**

Twenty-one relevant articles were identified, including 12 focused on vocal biomarkers and 9 addressing broader digital biomarkers. Vocal biomarker literature emphasized the lack of existing master protocols and the need for standardization. In contrast, digital biomarker research from organizations like the Digital Medicine Society offered structured frameworks applicable to voice research.

**Conclusion:**

There is currently no established master protocol for vocal biomarker development. This review highlights foundational elements necessary for future standardization efforts to support the clinical integration of vocal biomarkers in healthcare.

## 1 Introduction

Recent innovations in digital technology have shown promise for advancements in the medical field. Vocal biomarkers are of particular interest due to their noninvasive nature and broad applications. A vocal biomarker can be defined as a characteristic acoustic feature extracted from a voice sample (1). Through traditional acoustic analysis or artificial intelligence/machine learning techniques, these biomarkers can be associated with conditions that affect the voice. The integration of vocal biomarkers in a clinical setting is an emerging field of study. The primary aim of this review is to highlight the need for master protocols in vocal biomarker development, a rapidly growing field with potential applications in telemedicine, early disease detection, and personalized healthcare.

Using vocal biomarkers to monitor health offers several advantages over traditional diagnostic methods. The potential for integration into smartphones and other handheld devices means the ability for a diagnosis without ever seeing a provider (2). This has significant implications for global health, particularly in regions where healthcare access is limited. Different conditions also affect the voice in unique ways, meaning a comprehensive biomarker database would allow for a variety of conditions to be detected. Still, the viability of vocal biomarkers depends on trustworthiness, defined not only by ethical sourcing (including transparency, consent, and fairness) but also by responsible research practices that ensure robustness, reproducibility, and scientific integrity (3).

Because the technology associated with vocal biomarkers is relatively new, its clinical applications have been limited to this date. There are multiple factors that currently prevent vocal biomarkers from becoming a standardized practice in clinical medicine. Many datasets are privatized, and the ones that are available are not large enough to develop meaningful biomarkers (4), reflecting a broader structural issue in which stakeholders and industry lack strong incentives to make their data or methodologies transparent (5). Another significant challenge, and the focus of this review, is the lack of standardization in data collection protocols, necessitating a master protocol for vocal biomarker development.

A master protocol is a predefined, standardized framework that guides the design and execution of multiple related clinical studies within a single overarching protocol. Unlike traditional trial designs, master protocols enable harmonized data collection, consistent methodologies, and improved comparability across studies, ultimately increasing the efficiency and reproducibility of research findings (6). These protocols are particularly valuable in emerging fields like vocal biomarker development, where methodological variability is a major barrier to clinical adoption. Establishing a robust, interdisciplinary framework for vocal biomarkers would enable researchers to systematically evaluate biomarker validity, optimize data collection methodologies, and accelerate clinical translation.

Vocal biomarkers are a subset of digital biomarkers, which can be broadly defined as measurable characteristics collected through digital technologies to monitor normal biological processes (7). In addition to voice, digital biomarkers include metrics such as heart rate, sleep patterns, and gait. These biomarkers are particularly relevant to this study, as they provide a framework for comparison with existing research on vocal biomarkers. Organizations like the Digital Medicine Society (DiMe) have established rigorous guidelines for digital biomarker development, which can inform and strengthen the approach to vocal biomarker research. By leveraging these insights, more robust recommendations can be made for advancing vocal biomarker development.

This narrative review will begin by evaluating a set of studies that discuss the current state of standardization in vocal biomarker development, looking for gaps in the literature and recommendations for future research. Next, proposed master protocols for vocal biomarker development will be examined, comparing strengths and weaknesses and determining if a true master protocol exists. Finally, insights from the literature on digital biomarker development will be extracted to provide recommendations for voice protocol development.

## 2 Materials and methods

This narrative review examines existing literature on vocal biomarker development and the feasibility of a master protocol for standardization. A structured literature search was conducted, and findings were synthesized using a narrative thematic analysis to identify key trends and gaps in the field of vocal biomarker development.

A comprehensive literature search was conducted using PubMed for peer-reviewed articles. The search targeted studies related to vocal biomarkers and master protocols. Search terms included combinations of “vocal biomarkers” or “voice biomarkers” with “biomarker development” or “acoustic analysis”, as well as “biometric monitoring” with “clinical trials” or “standardization”. Additional searches included “master protocol” or “protocol standardization” in conjunction with “voice” or “speech”. The search was restricted to articles published in English within the last ten years to ensure relevance to current technological advancements.

To provide a relevant comparison for vocal biomarker development, a similar approach was taken using keyword searches and incorporating literature from the more established field of digital biomarkers. Given the broader availability of literature on digital biomarkers, multiple standardized protocols exist, allowing for a comparative analysis between best practices in digital biomarker development and the evolving methodologies in vocal biomarker research. By drawing insights from these well-established protocols, this review contextualizes the current landscape of vocal biomarkers, highlighting areas where methodological approaches align and where gaps remain.

Studies were selected based on predefined eligibility criteria. Articles were included if they investigated the development or clinical application of vocal biomarkers, addressed protocol standardization or methodological challenges, or provided insights from digital biomarker research applicable to vocal biomarkers. Studies that lacked direct relevance, focused exclusively on technical aspects of signal processing without clinical implications, or were published before 2015 were excluded. Non-English publications and duplicate studies were also removed. The selection process involved an initial screening of titles and abstracts to determine relevance, followed by a full-text review of eligible articles.

A narrative review was chosen over a systematic review due to the relatively limited body of research available on master protocols specific to vocal biomarker development. Since this is an emerging field, a broader and more flexible approach was necessary to capture key themes, methodological challenges, and gaps in the literature. Unlike a systematic review, which adheres to strict inclusion and exclusion criteria, a narrative review allows for a more comprehensive synthesis of findings, accommodating diverse sources and perspectives. Additionally, because this review integrates literature from both vocal and digital biomarker development, a flexible methodology was essential to ensure that relevant research from adjacent fields could be considered.

## 3 Findings

Following the literature search, a total of 21 articles were identified. After a detailed analysis and synthesis, these papers were categorized into three primary themes. The first category, variability in vocal biomarker development, includes five articles that compare and contrast the methodologies used across different study settings, primarily through review articles. The second category, proposed protocols, consists of seven articles that represent the closest examples of a master protocol for vocal biomarker development, covering a range of conditions and methodological approaches. The final category, insights from digital biomarkers, contains nine articles that highlight key findings from digital biomarker research, offering insights that are particularly relevant to vocal biomarker development.

### 3.1 Variability in vocal biomarker development

The majority of articles in this category highlighted a lack of standardization as a major barrier to cross-study comparison and implementing vocal biomarkers into clinical settings. Without standardized approaches to data collection, feature extraction, and model validation, the reproducibility of findings remains limited. This variability undermines confidence in the reliability of vocal biomarkers and creates significant barriers to regulatory approval and integration into clinical workflows. A master protocol would provide a unified framework to overcome these issues and ensure consistent, high-quality, trustworthy biomarker development.

Several studies illustrate the practical consequences of this inconsistency. A scoping review of voice biomarker applications in pediatric populations found variability across studies as a significant limitation, especially when it comes to the development of biomarker models. The authors explicitly called for the standardization of these methods (8). This sentiment was echoed by Bensoussan et al, citing a “lack of standards” as a barrier to comparing studies and pooling data (4). Even studies demonstrating the effectiveness of vocal biomarkers in disease detection emphasize the need for further validation before clinical implementation. In describing their work finding correlations between certain conditions and vocal biomarkers, Sara JD et al. emphasized the need for further standardization not only to increase clinical applicability but enhance diagnostic accuracy as well (1). Without a standardized framework to guide data acquisition, preprocessing, and analysis, the field risks producing biomarkers that are non-reproducible, biased, and clinically unreliable.

Idrisoglu et al. conducted a more quantitative analysis on variability in vocal biomarker development, systematically reviewing literature on machine learning techniques to diagnose voice-affecting conditions. There were several key findings, including no single dominant machine learning technique being used. The most commonly utilized ML technique, support vector machines, were employed in only 35.2% of studies. Many datasets were also unbalanced and did not include “additional data in conjunction with voice features” (9). This lack of methodological consistency highlights a fragmented research landscape, making it difficult to evaluate which techniques are most effective across different datasets. These limitations reduce the effectiveness of developed models and prevent their generalization to broader populations.

While data collection significantly impacts vocal biomarker quality, additional factors also contribute to variability. After data collection, audio must be compressed before vocal biomarkers can be extracted. An analysis of 17,298 uncompressed voice samples found that the audio compression algorithm used, such as MP3 vs. M4A vs. WMA, affected features crucial for vocal biomarker detection, compromising accuracy. For instance, jitter and shimmer, which are commonly used in clinical voice assessments, were distorted in compressed formats, leading to decreased sensitivity and specificity in biomarker detection (10). This finding suggests that without uniform guidelines on recording and compression methods, researchers may develop biomarkers that are not transferable across platforms or clinical settings.

Taken together, these studies highlight the need for a master protocol in order to standardize the way vocal biomarkers are built and extracted. Filling this gap in the literature would give researchers a set of guidelines to follow that includes data collection, audio compression, and eventually clinical implantation. Increased standardization in this field would not only make the process of building a vocal biomarker easier but also improve the accuracy of the biomarker itself to aid in diagnoses.

### 3.2 Proposed protocols

Given the widespread variability in vocal biomarker development, this review next examines whether existing literature has proposed a comprehensive master protocol. After reviewing the seven articles from this category, it was determined that no master protocol currently exists for vocal biomarker development. However, collectively, these articles outline important components that could inform the creation of one. These elements include guidelines for data collection, preprocessing, feature extraction, and clinical integration.

Standardized voice data collection procedures are among the most frequently cited needs. Multiple papers propose structured protocols for consistent microphone placement, reduction of ambient noise, and specific speaking tasks such as sustained phonation, read speech, and spontaneous speech to reduce recording variability and improve cross-study comparability (2, 11). Similarly, Sara et al. emphasize the importance of defining the recording environment and capturing multiple speech types to develop clinically relevant features (1).

Preprocessing and quality control of audio recordings also emerged as key steps. These include standardizing sampling rates, applying consistent normalization protocols, and filtering background noise prior to feature extraction (1). Multiple papers propose the extraction of both acoustic features, such as jitter, shimmer, and mel-frequency cepstral coefficients (MFCCs), and linguistic features, including speech rate and pause duration, to provide a holistic view of vocal health. Annotation of voice samples with clinically verified outcomes, such as diagnosis or symptom severity, is also emphasized as essential for training supervised learning models (2, 9, 11).

Fewer articles provide detailed clinical integration plans, but several acknowledge the importance of outlining how vocal biomarkers will be used in practice. Proposed uses include integration into telemedicine platforms, smartphone-based screening tools, and remote monitoring systems (12). Applications will require careful consideration of user interface design, regulatory compliance, and compatibility with existing clinical systems (2, 4).

**Figure 1:**
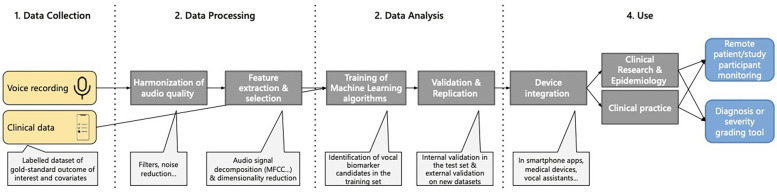
Pipeline for identification of vocal biomarkers. Reproduced with permission from “Pipeline for vocal biomarker identification, from research to practice” by Guy Fagherazzi, Aurélie Fischer, Muhannad Ismael and Vladimir Despotovic, licensed under CC-BY-NC.

Although no single protocol contains all of these components, their overlap reveals a growing consensus on what a robust master protocol might include. For example, Fagherazzi et al. outline a four-step pipeline of data collection, processing, analysis, and use that aligns closely with these findings (Figure 1). Sara et al. similarly describe a trajectory from voice recording to integration into clinical practice (1).

A true master protocol in this field, among other criteria, should cover everything from data collection to clinical integration, be applicable to multiple conditions, and be specific to vocal biomarkers. In the current literature, there is no paper that explicitly proposes a “master protocol” for vocal biomarker development, much less one that has been agreed upon by a panel of experts. Most protocols are not comprehensive enough to guide researchers working with varying recording conditions, participant demographics, and more.

### 3.3 Insights from digital biomarkers

In the broader context of digital biomarker research, vocal biomarkers represent one category among several modalities used for health monitoring. The Digital Medicine Society (DiMe) has published extensively on their development and integration. One of their most important resources, the V3 framework, has been cited over 250 times and leveraged by the FDA, NIH, and more. This protocol establishes a structured approach for assessing Biometric Monitoring Technologies (BioMeTs), which refer to the devices and systems digital biomarkers are extracted from (13). By applying the V3 framework to a digital biomarker, one can ensure its accuracy and clinical utility.

**Figure 2:**
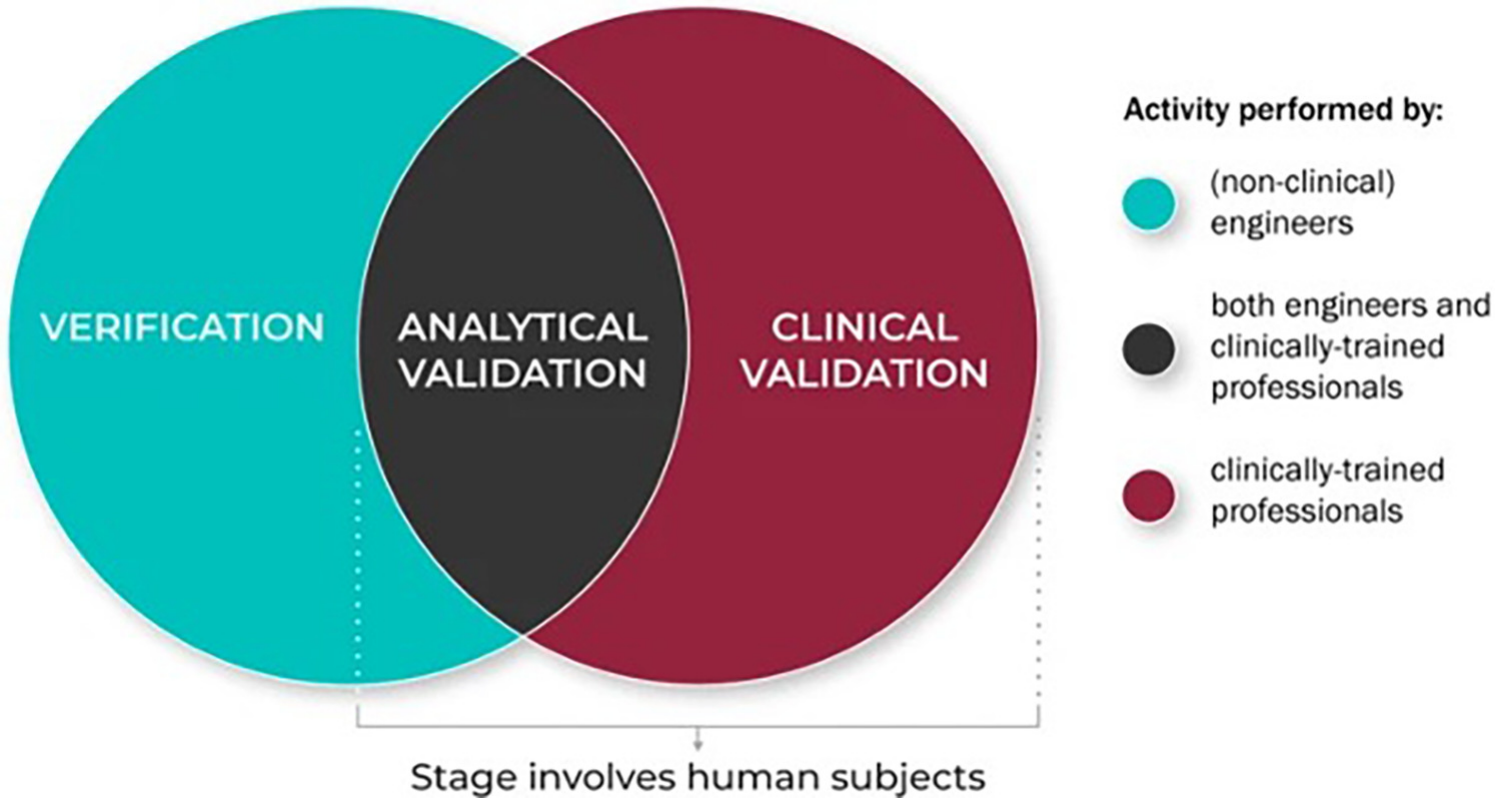
The role of different disciplines in the V3 process. Reproduced with permission from “The role of the different disciplinary experts in the V3 process: Verification, analytical validation, and clinical validation processes are typically conducted by experts across disciplines and domains” by Jennifer C. Goldsack, Andrea Coravos, Jessie P. Bakker, Brinnae Bent, Ariel V. Dowling, Cheryl Fitzer-Attas, Alan Godfrey, Job G. Godino, Ninad Gujar, Elena Izmailova, Christine Manta, Barry Peterson, Benjamin Vandendriessche, William A. Wood, Ke Will Wang and Jessilyn Dunn, licensed under CC BY 4.0.

The V3 framework consists of three components: verification, analytical validation, and clinical validation (Figure 2). This protocol is unique because it is multidisciplinary in nature: each component is carried out by a different set of professionals. Verification ensures that microphones and sensors capture high-fidelity signals without distortion and is done by non-clinical engineers (13). For vocal biomarkers, this would involve testing microphones under various conditions (quiet, noisy, different distances) to determine fidelity and consistency. Establishing a reference microphone type and placement standard, similar to guidelines proposed by Awan et al, could serve this role (14).

Clinical validation demonstrates that extracted vocal biomarkers correlate with health outcomes and is done by clinically trained professionals. Vocal biomarkers would need to demonstrate a robust association with specific health outcomes, such as disease severity, progression, or response to treatment. Integration into ongoing clinical trials provides an opportunity to validate vocal biomarkers in real-world, regulated environments while capturing diverse patient populations (15).

Finally, analytical validation confirms that voice processing algorithms reliably extract acoustic features linked to clinical conditions. This is done by both non-clinical engineers and clinically trained professionals in conjunction (13). For vocal biomarkers, voice-specific algorithms for acoustic feature extraction would need to demonstrate consistent performance across diverse datasets. This includes reproducibility of features across recording sessions and platforms.

While the framework itself has been explored considerably, its applications to vocal biomarkers specifically have not. A review paper on speech-based biomarkers found that “no speech measure has yet been comprehensively evaluated across all three categories” (16). This is where a master protocol comes in to bridge the gap. By outlining in detail the necessary steps, vocal biomarkers can be validated in a rigorous and clinically significant manner. The personnel of speech language pathologists and laryngologists on the clinical side with acoustic experts and engineers on the non-clinical side make the V3 framework a natural addition to a master protocol on vocal biomarker development.

The V3 framework is not the only outline for digital biomarker development. Another relevant contribution is the DACIA framework, which outlines five steps to develop digital biomarkers from wearable sensor data. This proposal combines guidance from the FDA and Digital Medicine Society to provide an outline that prioritizes stakeholder involvement in each step (17). Vocal biomarker research could adopt a similar structure by involving not only data scientists and clinicians, but also linguists, ethicists, and patient advocacy groups in protocol design.

Other considerations from digital biomarker research include a master protocol for the development and validation of gait. This protocol illustrates how a physical sensor (lumbar accelerometer) can be paired with standardized data analysis procedures to create a clinically validated digital biomarker (18). Analogously, vocal biomarkers could be paired with low-cost hardware such as headset microphones to create scalable voice-based tools for remote diagnostics.

### 3.4 Limitations

This review is subject to multiple limitations. As a narrative review, it does not provide a systematic or meta-analytic evaluation of the literature, meaning findings may be influenced by selection bias. Additionally, the field of vocal biomarker research is evolving rapidly, and new methodologies not included in this review may emerge.

## 4 Conclusion

The findings of this review highlight significant variability in vocal biomarker development, with major inconsistencies in data collection methods, signal processing, and clinical validation. These inconsistencies not only limit the reproducibility of research but also hinder the integration of vocal biomarkers into clinical practice. Despite increasing interest in voice as a digital biomarker, the absence of a comprehensive, standardized master protocol has prevented the field from achieving the same level of methodological rigor seen in other digital biomarker domains.

Although partial frameworks for vocal biomarker development exist, including proposed pipelines for data collection, processing, and clinical application, none provide a fully integrated approach that ensures standardization across different research and clinical settings. Existing studies provide important foundational insights, but they lack specific guidelines for recording standardization, model validation, and regulatory compliance. They also rarely incorporate governance mechanisms to ensure transparency, accountability, and the responsible acquisition of patient acoustic data and development of vocal biomarkers.

Insights from digital biomarker research offer promising directions for addressing these challenges. The V3 framework and DACIA framework could serve as a model for vocal biomarker development, but alone they are not sufficient. A vocal biomarker master protocol must be tailored to the unique challenges of voice-based diagnostics, including environmental variability, microphone standardization, and speech processing techniques.

Future research should focus on developing and validating a comprehensive master protocol that integrates best practices from digital biomarker research, clinical voice assessment, and computational modeling. Such a protocol should include clear guidelines for data acquisition, standardized preprocessing methods, validation strategies, and regulatory considerations to ensure that vocal biomarkers meet the necessary criteria for clinical implementation. Additionally, multi-institutional collaborations will be essential for developing a protocol that is widely accepted and applicable across diverse populations and clinical settings. Trustworthiness should also be embedded as a core design principle, ensuring transparency, ethical data practices, and accountability throughout the research and deployment pipeline.

Ultimately, the standardization of vocal biomarker development through a validated master protocol has the potential to revolutionize clinical diagnostics by enabling non-invasive, cost-effective, and scalable disease detection. As advancements in artificial intelligence, machine learning, and digital health continue to expand, the establishment of a unified framework for vocal biomarker research will be a key step toward realizing the full clinical potential of voice-based diagnostics.
